# Targeting Investigation and Treatment in Type 2 Myocardial Infarction: A Pilot Randomized Controlled Trial

**DOI:** 10.1016/j.jacadv.2025.101738

**Published:** 2025-04-04

**Authors:** Caelan Taggart, Amy Ferry, Stephanie Barker, Kelly Williams, Grace Souter, Anda Bularga, Ryan Wereski, Michael J. McDermott, Michelle C. Williams, Jasper Boeddinghaus, Christopher White, Jagdeep S. Singh, Keith Boath, Takeshi Fujisawa, Christopher Tuck, Anny Briola, Steff Lewis, Atul Anand, Marc R. Dweck, David E. Newby, Rustam Al-Shahi Salman, Nicholas L. Mills, Andrew R. Chapman

**Affiliations:** a BHF Centre of Research Excellence, University of Edinburgh, Edinburgh, United Kingdom; b Edinburgh Clinical Trials Unit, University of Edinburgh, Edinburgh, United Kingdom; c Cardiovascular Research Institute, University Hospital Basel, University of Basel, Basel, Switzerland; d Usher Institute, University of Edinburgh, Edinburgh, United Kingdom; e Victoria Hospital, Kirkcaldy, Scotland; f Centre for Clinical Brain Sciences, University of Edinburgh, Edinburgh, United Kingdom

**Keywords:** coronary angiography, echocardiography, secondary prevention, type 2 myocardial infarction, universal definition

## Abstract

**Background:**

Type 2 myocardial infarction occurs in the absence of atherothrombosis, due to myocardial oxygen supply or demand imbalance, often during another acute illness. It is common and associated with poor clinical outcomes. No randomized controlled trials are available to guide investigation or treatment.

**Objectives:**

The authors assessed the feasibility of implementing a complex intervention of investigation and treatment for coronary and structural heart disease in patients with type 2 myocardial infarction.

**Methods:**

A pilot phase of a prospective randomized controlled trial was conducted. Process outcomes included the proportion of eligible patients approached, consented, and randomized. Adherence was defined as the number of recommended investigations and treatments administered at 90 days. Qualitative interviews explored reasons for participation and patient experience.

**Results:**

Between November 2022 and November 2023, 4,127 patients with increased cardiac troponin concentrations were screened across 3 sites, and 403 patients (10%) met inclusion criteria. One hundred and forty-three patients (35%) were eligible, 119 patients (83%) were approached, and 60 patients (42%, age 70 ± 10 years, 38% women) consented and randomized to the intervention (n = 28) or standard care (n = 32). Follow-up was complete in all participants. Adherence to recommendations was 90.7% (95% CI: 85.3%-96.1%). Patients highlighted variation in communication of the diagnosis and in trial investigation and management recommendations were potential barriers to participation.

**Conclusions:**

It is feasible to recruit and randomize patients with type 2 myocardial infarction to a complex intervention targeting coronary or structural heart disease. A multicenter trial with an optimized intervention is now required to inform practice.

Type 2 myocardial infarction (MI) occurs when there is an imbalance between myocardial oxygen supply or unmet need in myocardial oxygen demand, without evidence of atherothrombosis.^1^ These events often happen in the context of physiological stress from another acute illness.^2^ Patients with type 2 MI have significantly worse clinical outcomes than those with type 1 MI. As few as 30% of patients are alive at 5 years after diagnosis,^3^, ^4^, ^5^, ^6^ and risk factors for both type 1 and type 2 MI are similar, with age, hyperlipidemia, diabetes mellitus, abnormal renal function, and known coronary disease independent predictors of both.^7^^,^^8^ Patients with type 2 MI have a six-fold increased rate of recurrent type 2 events compared to those without, suggesting a missed opportunity to address the underlying pathology.^8^

While there are clear treatment strategies in type 1 MI which are proven to improve clinical outcomes, there are no completed randomized controlled trials to guide practice in type 2 MI. We previously hypothesized that patients with type 2 MI have a high prevalence of unrecognized coronary artery disease or left ventricular impairment (DEMAND-MI [Determining the Mechanism of Myocardial Injury and Role of Coronary Disease in Type 2 Myocardial Infarction], NCT03338504), and these are in part responsible for poor clinical outcomes. We found two-thirds of patients with type 2 MI had coronary artery disease and one-third had evidence of left ventricular impairment.^9^ These findings have since been validated in DEFINE-MI (Defining the Prevalence and Characteristics of Coronary Artery Disease Among Patients with Type 2 Myocardial Infarction using CT-FFR), where computed tomography (CT) coronary angiography identified coronary artery disease in 92% of patients, which was obstructive in 42% and previously unrecognized and untreated in almost all.^10^

We hypothesize that risk assessment and targeted investigation to guide secondary prevention for coronary artery disease and/or left ventricular impairment in patients with type 2 MI will reduce future MI, heart failure hospitalization, or cardiovascular death. We intend to test this approach in a large multicenter randomized controlled trial. However, previous efforts to randomize patients with type 2 MI have been unsuccessful as the diagnosis is challenging and often made retrospectively in patients who are acutely unwell.^11^ We therefore first aimed to demonstrate in a pilot feasibility trial that patients with type 2 MI can be prospectively identified, recruited, and randomized in different health care settings to identify facilitators and barriers to recruitment and ensure acceptability of our intervention to patients and clinicians.

## Methods

### Study population

TARGET-Type 2 (TARGETing investigation and treatment in patients with Type 2 myocardial infarction) is the pilot phase of a PROBE (prospective randomized open-label with blinded endpoint evaluation) trial evaluating a complex intervention in patients with type 2 MI (NCT05419583). This study was approved by the North of Scotland Research Ethics Committee (REC: 22/NS/0085) and carried out in conjunction with the Edinburgh Clinical Trials Unit. A trial steering committee provided oversight with independent members including those with expertise in complex intervention trials and patient and public involvement and engagement (PPIE) representatives with lived experience of this condition.

### Sites and participants

Participants were recruited from 3 sites in Scotland: the Royal Infirmary of Edinburgh, Western General Hospital, and Victoria Hospital Kirkcaldy. Potential participants were identified through cardiac troponin concentrations, which were obtained as part of routine clinical care at the principal site (Royal Infirmary of Edinburgh). Following review of the electronic medical record, patients with acute myocardial injury (cardiac troponin concentration >99th centile) who met inclusion criteria for type 2 MI were entered into a screening log. If these patients were not eligible, reasons for exclusion were documented. In the second and third sites (Western General Hospital and Victoria Hospital, Kirkcaldy), potential participants were identified by direct referral to the research team.

### Inclusion and exclusion criteria

Inclusion criteria were based on the diagnostic criteria for type 2 MI proposed in the Fourth Universal Definition of Myocardial Infarction.^1^ These included: 1) symptoms of myocardial ischemia or signs of myocardial ischemia on 12-lead electrocardiogram (≥0.5 mm ST-segment depression in any 2 contiguous leads or new regional T-wave inversion); 2) acute myocardial injury defined as a clinically significant change in high-sensitivity cardiac troponin concentration with at least one value above the 99th centile upper reference limit or a single measurement if considered significantly elevated; and 3) documented evidence of myocardial oxygen supply (anemia, hypoxia, hypotension, bradycardia, tachycardia, arrhythmia) or demand (hypertension, left ventricular hypertrophy, valvular heart disease) imbalance. We did not mandate specific criteria for myocardial oxygen supply or demand imbalance in recognition that the threshold for ischemia may differ at an individual patient level.^12^ Cardiac troponin concentrations were measured using the Roche Elecsys high-sensitivity cardiac troponin assay (Roche Diagnostics). This assay has a limit of detection of 3 ng/L and an interassay coefficient of variation of 10% at 13 ng/L. The 99th centile is 9 ng/L for females and 16 ng/L for males.^13^ We excluded patients under 30 years who are less likely to benefit from cardiac imaging, patients who were unable to give informed consent, those on renal replacement therapy or with estimated glomerular filtration rate (eGFR) <30 mL/min, those with advanced frailty (based on Clinical Frailty Score ≥7^14^), those who were pregnant or breast feeding, those with ST-segment elevation on 12-lead electrocardiogram, those with a presumed or confirmed diagnosis of type 1 MI due to atherothrombosis, those with a confirmed coronary mechanism of type 2 MI (vasospasm, embolism, or spontaneous coronary artery dissection), and those previously randomized into the TARGET-Type 2 pilot study.

### Randomization

Patients were randomized 1:1 to the complex intervention or standard care using a web-based computer-generated randomization process developed by the Edinburgh Clinical Trials Unit using SQL and incorporated into our REDCap database. Randomization was stratified by the presence of: 1) known coronary artery disease; and 2) known left ventricular systolic dysfunction.

### Complex intervention: investigation

The complex intervention was a structured management plan to guide; 1) risk assessment; 2) targeted investigation; and 3) secondary preventative therapy delivered after consultation with a cardiologist. The intervention was individualized and delivered by a single principal investigator (consultant cardiologist) at each site. The cardiologist estimated if the pretest probability of coronary artery disease or left ventricular impairment was low, intermediate, or high based on symptoms, cardiovascular risk factors, previous medical history, and an evaluation of the presenting illness and initial assessment including a physical examination, routine blood tests, the 12-lead electrocardiogram, and chest x-ray. Where the pretest probability of coronary disease or left ventricular impairment was low, further investigation was not recommended. Where the pretest probability of coronary artery disease was intermediate, coronary computed tomography angiography was recommended and performed according to previously published methods and reported in line with SCCT (Society of Cardiac Computed Tomography) guidelines.^15^ Where the pretest probability of obstructive coronary disease was considered high or if the patient had ongoing symptoms of myocardial ischemia, invasive coronary angiography was recommended. Where the patient was known to have coronary artery disease, no further coronary imaging was recommended unless there was a clinical suspicion of worsening disease severity.

Where the pretest probability of left ventricular impairment was intermediate or high, a transthoracic echocardiogram was recommended and conducted in accordance with guidance from the British Society of Echocardiography. Where there was a suspicion of underlying cardiomyopathy or type 1 MI based on echocardiography, cardiovascular magnetic resonance and coronary imaging were considered (if not already performed) to identify areas of scar or to clarify the final diagnosis in line with usual recommendations for clinical practice. We aimed to deliver all investigations within 30 days.

### Complex intervention: treatment

Treatment was individualized and in line with clinical practice guidelines.^16^ In patients found to have coronary artery disease, aspirin or P_2_Y_12_ inhibitors and lipid-lowering therapy with statins were recommended. P_2_Y_12_ inhibitors were recommended in those with aspirin intolerance or previous stroke. In patients who underwent invasive coronary angiography, revascularization with percutaneous coronary intervention was recommended if there was evidence of recent plaque rupture or where high-risk features were present. Revascularization was also considered in patients with obstructive coronary artery disease who reported limiting anginal symptoms despite medical therapy. In patients with evidence of left ventricular impairment, additional therapies including beta-blockers, angiotensin-converting enzyme (ACE) or angiotensin receptor blocker or aldosterone/neprilysin inhibitors, mineralocorticoid receptor antagonists, and sodium-glucose cotransporter 2 inhibitors were recommended in line with guideline recommendations.

### Standard care

In order to document contemporary clinical practice in this feasibility trial, we did not restrict access to investigation or treatment in the standard care arm. Patients in the standard care arm received all investigation and treatment as recommended by the usual care team.

## Outcomes

This is a pilot feasibility trial which is not powered for clinical endpoints. Feasibility outcomes included the proportion of screened patients who were eligible, approached, consented, and randomized. Adherence was calculated as the proportion of recommendations and treatments administered at 90 days. All clinical endpoints including subsequent MI, hospitalization with heart failure, and death at 90 days were identified using electronic patient record review. A nested qualitative study was undertaken to understand patient motivation for participation and to identify potential barriers to recruitment.

### Follow-up and adjudication of myocardial infarction

We used electronic medical records to ensure complete follow-up for the study population from the date of the index admission to 90 days. Following discharge, all patients who reattended with suspected acute coronary syndrome were identified using an electronic screening tool embedded into routine clinical practice. In patients with at least one cardiac troponin concentration above the sex-specific 99th centile upper reference limit, the diagnosis of type 1 or type 2 MI or acute or chronic myocardial injury was adjudicated by a panel of cardiologists according to the Fourth Universal Definition of Myocardial Infarction. Abnormal renal function was defined in patients with an eGFR <60 mL/min/1.73 m^2^. A detailed summary of the adjudication procedures is provided in the Supplemental Appendix.

### Qualitative intervention and thematic analysis

Interviews were performed by a cardiology research nurse and trained qualitative researcher within 6 weeks of being invited to take part in the trial by telephone, face-to-face, or over video call (NHS Near Me). We explored views on the presentation of trial information, understanding of study processes (eg, randomization), the acceptability of study interventions, and reasons underlying decisions to consent or decline to participate. A topic guide developed with a patient and public involvement group with lived experience of type 2 MI was used to guide the interview (Supplemental Appendix). If clinician hesitation to randomize their patient was noted to be a barrier to recruitment, we planned dedicated interviews to explore this. Interviews were conducted until data saturation was achieved and additional interviews did not yield new insights.^17^ Data collection and analysis occurred concurrently. An interpretive approach was used to analyze data thematically,^18^ using abductive reasoning, which seeks to identify meaning from the accounts in iteration with prior knowledge from the field. Open coding was applied to transcripts, which were grouped into subcategories based on similarities and patterns identified. Data were managed using NVivo 12 software (QSR International) and presented as quotes from transcripts.

### Statistical analysis

As this was a pilot feasibility trial, no power calculation was undertaken, but consensus recommends the recruitment of between 40 and 72 patients.^19^ We aimed to recruit a total of 60 participants. Quantitative data are presented as mean ± SD or median (IQR) based on their distribution. For estimates of proportions, 95% CIs are provided for primary outcome measures as appropriate. Adherence to management recommendations at 90 days was calculated, reporting the number of investigations undertaken and the number of treatments prescribed as a proportion of those recommended, respectively. The frequency of efficacy and safety outcomes in both the intervention and the control arm are reported. All data were recorded in a prospective REDCap database held within a secure data environment. Qualitative data were analyzed using Braun and Clarks’ approach to thematic analysis.^18^ All statistical analysis was undertaken using SAS (Version 9.4, SAS Institute) or R (Version 4.4.1, The R Project for Statistical Computing). This trial has been reported in accordance with CONSORT (Consolidated Standards of Reporting Trials) guidelines.

## Results

### Trial population and feasibility of recruitment

**Figure 1:**
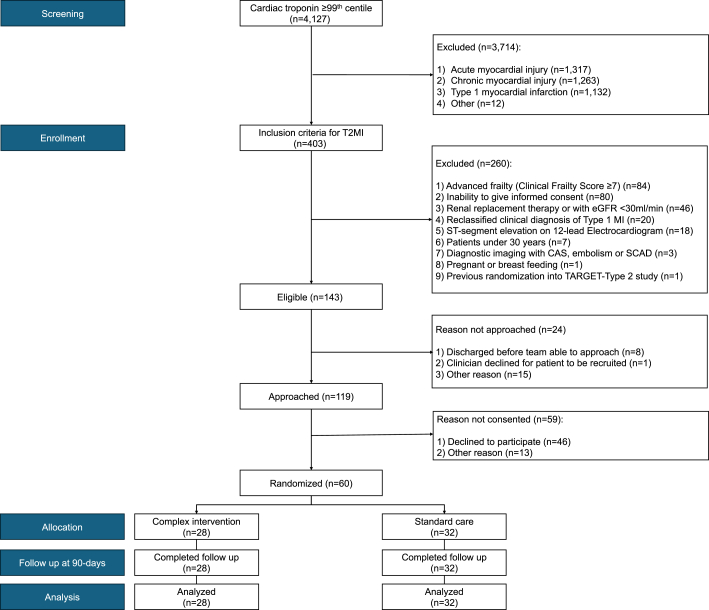
Trial Flow Diagram According to CONSORT Guidelines CAS = coronary artery spasm; CONSORT = Consolidated Standards of Reporting Trials; eGFR = estimated glomerular filtration rate; MI = myocardial infarction; SCAD = spontaneous coronary artery dissection; T2MI = type 2 myocardial infarction.

From November 2022 to November 2023, a total of 4,127 patients with elevation in cardiac troponin concentration ≥99th centile were screened across 3 participating sites, of whom 403 (9.8%) met inclusion criteria with a diagnosis of type 2 MI (Central Illustration). One hundred and forty-three patients (35%) were eligible, 119 patients (83%) were approached, and 60 patients (42%, age 70 [SD 10] years, 38% women) consented and randomized to the intervention (n = 28) or standard care (n = 32). A total of 260 (65%) of patients meeting inclusion criteria met one or more exclusion criteria, due to advanced frailty (32% [84/260]), inability to give informed consent due to illness severity (31% [80/260]), or renal replacement therapy/eGFR ≤30 mL/m^2^ (17% [46/260]). A trial flow diagram according to CONSORT guidelines details all reasons for exclusion and is provided in Figure 1.

**Central Illustration:**
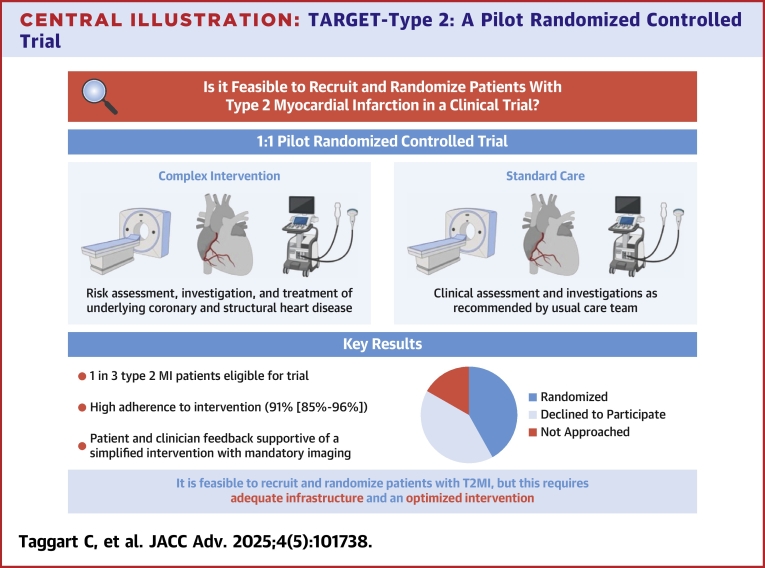
TARGET-Type 2: A Pilot Randomized Controlled Trial TARGET-type 2 was a pilot randomized controlled trial demonstrating it is feasible to recruit and randomize patients with type 2 myocardial infarction to a complex intervention targeting underlying coronary or structural heart disease. A main phase trial with an optimized intervention is now required to inform clinical practice. Abbreviations as in Figure 1.

In all patients, the adjudicated diagnosis was type 2 MI. The recruitment rate was 5 patients per month (Supplemental Figure 1), with the majority of participants recruited from one site (Supplemental Table 1). There was a high prevalence of known coronary artery disease (25% [15/60]), hypertension (43% [26/60]), diabetes mellitus (20% [12/60]), and chronic obstructive pulmonary disease (18% [11/60]).

Most participants experienced type 2 MI due to tachyarrhythmia (47% [28/60]) or hypoxemia (22% [14/60]), with 37% (22/60) of participants in atrial fibrillation or flutter at the time of presentation. Electrocardiographic evidence of myocardial ischemia was present in 75% (45/60) of participants. Most patients were tachycardic (median heart rate 135 beats/min, IQR: 105-180 beats/min, with 20% [12/60] of patients receiving supplemental oxygen therapy [Supplemental Table 2]).

Differences in baseline medication prescriptions were identified between groups, with more participants in the standard care arm compared to the intervention arm already established on an ACE inhibitor or angiotensin receptor blocker (50% [16/32] vs 32% [9/28]), antiplatelet therapy (38% [12/32] vs 25% [7/28]), and statin therapy (56% [18/32] vs 46% [13/28]) at the time of recruitment (Table 1).

**Table 1:** Baseline Characteristics of the Trial Population

	All Patients (N = 60)	Intervention (n = 28)	Standard Care (n = 32)
Age (y)	71 (65-79)	67 (63-77)	75 (67-80)
Female	23 (38)	8 (29)	15 (47)
Ethnicity			
White British	58 (97)	26 (93)	32 (100)
Asian British - Indian	1 (2)	1 (4)	0 (0)
Not specified	1 (2)	1 (4)	0 (0)
Medical history			
Known coronary artery disease	15 (25)	8 (29)	7 (22)
Previous MI	10 (17)	4 (14)	6 (19)
Previous PCI	10 (17)	6 (21)	4 (13)
Previous CABG	5 (8)	3 (11)	2 (6)
Known LV systolic dysfunction	8 (13)	3 (11)	5 (16)
Previous stroke	5 (8)	2 (7)	3 (9)
Hypertension	26 (43)	12 (43)	14 (44)
Diabetes mellitus	12 (20)	7 (25)	5 (16)
Chronic obstructive pulmonary disease	11 (18)	5 (18)	6 (19)
Current smoker	11 (18)	4 (14)	7 (22)
Family history of premature coronary disease	21 (35)	10 (36)	11 (34)
Mechanism of supply or demand imbalance			
Tachyarrhythmia	28 (47)	14 (50)	14 (44)
Hypoxia	14 (22)	7 (25)	6 (19)
Anemia	5 (8)	3 (11)	2 (6)
Hypotension	4 (7)	1 (4)	3 (10)
Hypertension	4 (7)	1 (4)	3 (10)
12-lead electrocardiogram			
Sinus	31 (52)	13 (46)	18 (56)
Atrial fibrillation	19 (32)	10 (36)	9 (28)
Flutter	3 (5)	1 (4)	2 (6)
Other	7 (11)	4 (14)	3 (9)
ST-elevation	8 (13)	5 (18)	3 (9)
ST-depression	30 (50)	16 (57)	14 (44)
T-wave inversion	29 (48)	13 (46)	16 (50)
New ischemic changes	45 (75)	24 (86)	21 (66)
Hematology and biochemistry at presentation			
Hemoglobin (g/L)	139 (120-150)	139 (116-156)	137 (121-148)
Creatinine (μmol/L)	89 (71-105)	88 (74-106)	90 (67-105)
eGFR >60 mL/min	42 (70)	20 (71)	22 (69)
Peak cardiac troponin T concentration (ng/L)	76 (41-177)	72 (45-172)	84 (33-187)
Medical therapies at presentation			
ACE inhibitor/ARB	25 (42)	9 (32)	16 (50)
Beta-blocker	30 (50)	14 (50)	16 (50)
Aspirin	16 (27)	6 (21)	10 (31)
P2Y12 inhibitor	3 (5)	1 (4)	2 (6)
SGLT2 inhibitor	5 (8)	2 (7)	3 (9)
Oral anticoagulant	18 (30)	8 (29)	10 (31)
Statin	31 (52)	13 (46)	18 (56)
Diuretics	14 (23)	6 (21)	8 (25)

Values are median (IQR) or n (%).

ACE = angiotensin-converting enzyme; ARB = angiotensin receptor blocker; CABG = coronary artery bypass graft; eGFR = estimated glomerular filtration rate; LV = left ventricle; MI = myocardial infarction; PCI = percutaneous coronary intervention; SGLT2 = sodium-glucose cotransporter 2.

### Efficacy and process outcomes

**Figure 2:**
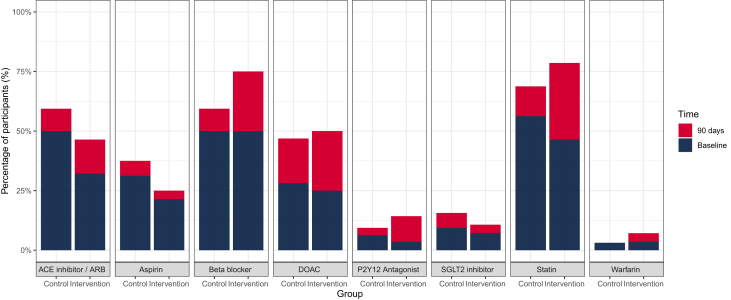
A Comparison of Medications Received at 90 Days Post Randomization in the Control and the Intervention Arm The dark shaded area represents the proportion of medications which were prescribed to patients at the time of randomization, with the red shaded area indicating the proportion of additional new medications prescribed at 90 days. ACE = angiotensin-converting enzyme; ARB = angiotensin receptor blocker; DOAC = direct oral anticoagulant; SGLT2 = sodium-glucose cotransporter 2.

In the intervention arm, CT or invasive coronary angiography was recommended in 50% (14/28) of patients and echocardiography in 79% (22/28) of patients, leading to new prescriptions for antiplatelet or anticoagulant therapy in 54% (15/28) of patients, ACE inhibitors or beta-blockers in 43% (12/28) and statin therapy in 39% (11/28) patients (Figure 2). Adherence to recommendations was 90.7% (95% CI: 85.3% to 96.1%) at 90 days. Nonadherence primarily occurred due to delay in treatments/investigations or patient noncompliance (Table 2), resulting in fewer investigations achieved by 90 days (Table 3).

**Table 2:** Investigation, Treatment Recommendations, and Adherence in the Intervention Arm (n = 28)

Recommended investigations	
CT coronary angiography	8 (29)
Invasive coronary angiography	6 (21)
Echocardiography	22 (79)
Cardiac magnetic resonance imaging	0 (0)
Number of recommended investigations per patient	
0	3 (11)
1	14 (50)
2	11 (39)
Recommended medications	
Antiplatelet	10 (36)
Anticoagulant	16 (57)
Statin	23 (82)
Beta-blocker	23 (82)
Heart failure therapy	6 (21)
Number of recommended medications per patient	
1	3 (11)
2	5 (18)
3	16 (57)
≥3	4 (14)
Total number of recommendations made (mean [SD])	3.7 (1.2)
Adherence (mean [SD])	90.7 (14.6)
Reason for nonadherence	
Primary caregiver not in agreement	1 (11)
Patient noncompliance	4 (44)
Delay in investigation or treatment	4 (44)

Values are n (%).

**Table 3:** Observed Investigation and Treatment Delivered at 90 Days

	All Patients (N = 60)	Intervention (n = 28)	Standard Care (n = 32)
Investigations			
CT coronary angiography	6 (10)	5 (18)	1 (1)
Invasive coronary angiography	13 (22)	5 (18)	8 (25)
Echocardiography	37 (62)	20 (71)	17 (53)
Cardiac MRI	0 (0)	0 (0)	0 (0)
New prescription of medication administered at 90 d			
Aspirin	8 (13)	3 (11)	5 (16)
P2Y12	6 (10)	3 (11)	3 (9)
Any antiplatelet	14 (23)	6 (21)	8 (25)
Anticoagulant	17 (28)	9 (32)	8 (25)
Statin	18 (30)	11 (39)	7 (22)
Beta-blocker	13 (22)	8 (29)	5 (16)
ACE inhibitor	7 (12)	4 (14)	3 (9)
All medication received at 90 d			
Aspirin	19 (32)	7 (25)	12 (38)
P2Y12	7 (12)	4 (14)	3 (9)
Any antiplatelet	26 (43)	11 (39)	15 (47)
Anticoagulant	32 (53)	16 (57)	16 (50)
Statin	44 (73)	22 (79)	22 (69)
Beta-blocker	40 (66)	21 (75)	19 (59)
ACE inhibitor	26 (43)	13 (46)	13 (41)

Values are n (%).

Abbreviations as in Table 1.

In the standard care arm, at 90 days post randomization, CT or invasive coronary angiography was undertaken in 28% (9/32) patients, with echocardiography in 53% (17/32) patients, leading to new prescriptions for antiplatelet or anticoagulant therapy in 50% (16/32), ACE inhibitors or beta-blockers in 25% (8/32) and statin therapy in 22% (7/32) of patients (Table 3).

### Safety outcomes

At 90-day follow up, death from any cause occurred in one patient in the intervention arm and 3 patients in the standard care arm. In the intervention arm, there were no deaths from a cardiovascular cause, one readmission with heart failure, and one subsequent type 2 MI (7% [2/28]). In the standard care arm, there were 3 deaths from a cardiovascular cause and one readmission with heart failure (13% [4/32]). There were no bleeding or unscheduled revascularization events (Table 4).

**Table 4:** Primary and Secondary Outcomes at 90 Days Post Randomization

	All Patients (N = 60)	Intervention (n = 28)	Standard Care (n = 32)
Myocardial infarction, heart failure hospitalization, or cardiovascular death	6 (10)	2 (7)	4 (13)
Death			
All-cause death	4 (7)	1 (4)	3 (10)
Cardiovascular death	3 (5)	0 (0)	3 (10)
Cardiac death	3 (5)	0 (0)	3 (10)
Non cardiovascular death	1 (2)	1 (4)	0 (0)
Heart failure hospitalization	2 (3)	1 (4)	1 (3)
Myocardial infarction	1 (2)	1 (4)	0 (0)
Stroke	1 (2)	1 (4)	0 (0)
Coronary revascularization	2 (3)	1 (4)	1 (3)
Unscheduled revascularization	0 (0)	0 (0)	0 (0)
Major bleeding	0 (0)	0 (0)	0 (0)

Values are n (%).

### Qualitative outcomes

The first 21 eligible trial participants were approached to participate in the qualitative study, of whom 19 agreed to participate. This group included 17 patients who were enrolled in the pilot trial and 2 patients who declined to participate. The final sample size was 13 patients; 2 were not contactable, one was readmitted to hospital, and one declined participation at the time of phone call. We evaluated participants motivation for involvement, their views on randomization, their understanding of diagnosis, and reasons for nonparticipation. Following early analysis, an emerging theme of “understanding of diagnosis” emerged, and 6 participants attended a focus group to explore this in more detail. As all clinicians were supportive of the trial and willing for their patients to be approached and randomized, no clinician interviews were conducted.

The majority participated in the trial for altruistic reasons and wanted to contribute to advancing health care knowledge. Some recognized that participation may lead to additional care which may be of benefit, and others participated due to encouragement by the usual care team. While participants randomized to the intervention arm were universally positive, those randomized to standard care were sometimes disappointed. Importantly, as this was a complex intervention trial, some patients were confused as to what their involvement in the trial would entail. The participant information sheet described all possible interventions that could be considered, and patients felt a clearly described single intervention would be helpful.

Our focus group identified variable use of the term “type 2 myocardial infarction” in clinical practice, and for some it was the research team and not the clinical team who introduced this term. This influenced patient processing of information and the emotional and practical impacts of the diagnosis. Furthermore, participants identified that there were no educational resources available for patients either in the hospital or online. A summary of key conclusions is provided in Supplemental Table 3, with representative quotes available in the Supplemental Appendix.

## Discussion

TARGET-Type 2 was a pilot feasibility randomized controlled trial which aimed to assess recruitment and randomization of patients with type 2 MI into a complex intervention targeting underlying coronary heart disease and left ventricular impairment. Through a qualitative study, we aimed to evaluate the acceptability of our intervention to patients and clinicians, to optimize our trial intervention, and to identify any barriers to recruitment. We report several findings which will be of benefit to the design of future clinical trials in patients with type 2 MI.

Firstly, we have demonstrated it is possible to prospectively identify consecutive patients with type 2 MI through screening of routine electronic health care records. Secondly, despite increasing age and comorbidity, patients with type 2 MI were willing and able to be consented and randomized in a clinical trial. Thirdly, we were able to deliver a complex intervention of recommendations for further investigation and treatment with high adherence at 90 days and no signal of harm. Finally, through a targeted qualitative intervention, we have gained important insight into the acceptability and possible limitations of our proposed intervention which will allow us to refine our study design for a main phase trial.

Although the nomenclature of type 2 MI originates in the 2007 Universal Definition,^20^ to date, no dedicated clinical trials have been successfully undertaken, demonstrating the challenges in recruitment in this heterogeneous, multimorbid population. One single-center randomized controlled trial aimed to investigate the effect of rivaroxaban or placebo on a composite outcome of MI, stroke, or death at 2 years.^11^ The trial did not complete due to a lack of trial infrastructure and insufficient resources to deliver the intervention. A second single-center randomized controlled trial planned to determine if coronary revascularization could reduce infarct size in patients with type 2 MI but was withdrawn prior to starting recruitment. The ACT-2 (Appropriateness of Coronary investigation in myocardial injury and Type 2 myocardial infarction; ACTRN 12618000378224) randomized controlled trial aims to enroll patients with secondary myocardial injury or infarction and randomize them to coronary angiography and secondary prevention vs standard care.^21^ The ACT-2 trial is powered to detect a 20% relative risk reduction in all-cause death at 2 years. It mandates strict inclusion criteria for myocardial oxygen supply or demand imbalance and began enrollment in 2019 but has yet to complete (personal communication). In this context, we anticipated there would be challenges in the delivery of a randomized controlled trial in this population and elected to undertake a feasibility trial to optimize our approach.

One of the major challenges in the adoption of type 2 MI as a diagnostic term in clinical practice is the heterogeneity within the current classification, and several revisions to the Universal Definition have been proposed.^22^, ^23^, ^24^, ^25^ The lack of adoption in clinical practice reflects uncertainty surrounding the utility of this diagnostic term, and further research is required to identify clinical features which are related to future cardiovascular outcomes. At present, there are few circumstances in which patients with type 2 MI would benefit from a single strategy for investigation and treatment, and to attempt to navigate this heterogeneity, we designed a complex intervention. By targeting underlying coronary artery disease and left ventricular impairment, the focus changes from treatment of the primary etiology—the current standard of care—to identification and treatment of the underlying disease with evidence-based therapies. This strategy has the potential to modify the risk of future type 1 or type 2 MI and reduce heart failure hospitalization or death from cardiovascular disease.

TARGET-Type 2 is the first dedicated clinical trial to successfully recruit and randomize patients with type 2 MI. In line with guidance from the Medical Research Council on the evaluation of complex intervention trials,^26^ we have considered all aspects of our study design in conjunction with our trial steering committee and PPIE group. We achieved a recruitment rate of just over 5 patients per month, with most participants originating from a single site where the chief investigator and research team were based. Although consecutive patient screening using electronic patient records was the intended approach in all centers, there was insufficient funding and resource allocation to support this trial activity in one site, where this reverted to individual patient referral to the local principal investigator. The recruitment rate achieved reflects their availability. At the principal site where consecutive patients with myocardial injury were screened, one in 10 met the Universal Definition criteria for type 2 MI, which is consistent with previous observations.^3^ Future multicenter clinical trials need adequate resource to enable screening of consecutive patients with acute myocardial injury to maximize translation from screened to recruited participants.

We had several exclusion criteria, which led to over half of eligible participants being excluded, which may pose challenges for the delivery of a main phase trial. We excluded 20% of eligible participants who were unable to give informed consent at the time of first approach due to illness severity. We discussed this challenge with a local critical care PPIE group, who recommended future studies should consider maintaining an active screening log to approach these patients after recovery. We excluded 21% of eligible participants with a Clinical Frailty Score of ≥7 (ie, fully dependent). This is likely to have been biased by illness severity during the initial assessment, and an evaluation of premorbid frailty is likely to be more informative. An alternative approach would be to restrict recruitment to those who are independent and mobile at the time of discharge where life expectancy is >12 months. Taken together, these measures may increase the number of potential participants for inclusion.

To explore feasibility and retain flexibility in the design of our intervention for a main phase trial, investigations were individualized. We did not mandate invasive or CT coronary angiography or echocardiography in the intervention arm, and in order to document practice in the standard care arm, we did not limit clinicians in their choice of investigation or treatment. This translated to modest differences in recommendations for investigation between patients in the intervention and the standard care arm. Although coronary angiography is not recommended in any clinical guideline for type 2 MI, the observed rates in the standard care arm were higher than anticipated when compared to prior observational data.^3^ This may be due to an increased awareness of type 2 MI within the principal site as a result of conducting the trial. The lack of significant differences in investigation received in the intervention arm likely contributed to similar rates of medical therapy being prescribed at 90 days, and while this comparison is underpowered and not the intention of this pilot trial, this may also in part reflect imbalance in randomization due to small numbers of trial participants as some differences in baseline treatment were observed.

Variability in the trial intervention was raised by both patients and clinicians as a potential barrier to recruitment, and we agreed with our PPIE group that a future trial should mandate cardiac imaging. Potential participants in whom the attending cardiologist feels invasive or CT coronary angiography is essential for diagnostic purposes would not be eligible for recruitment. This approach will maximize differences between trial populations and allow us to evaluate the safety and efficacy of systematic cardiac imaging in patients with type 2 MI where there is clinical equipoise.

Further feedback suggested that participants found the diagnostic terminology around type 2 MI confusing and they received inconsistent information from clinicians. At present, there are no dedicated patient-facing resources available which can contribute to fear and uncertainty around future prognosis when patients seek information from the internet. Therefore, one focus for optimizing patient care in type 2 MI must be improved access to patient-facing resources.

While TARGET-Type 2 incorporated the targeting of secondary prevention, an alternative strategy for clinical trials in type 2 MI is to target the etiology of specific disease phenotypes. The MINT (Myocardial Ischaemia and Transfusion) trial evaluated a restrictive vs liberal transfusion strategy in patients with MI. Most of the participants recruited (55.8%) had type 2 MI, but in this subgroup, no differences in MI or death were observed at 30 days.^27^^,^^28^ While this analysis was prespecified, these diagnoses were not adjudicated and the trial was not powered for this comparison. Prospective trials evaluating specific therapeutic strategies with the aim of reducing ischemic burden in the acute phase of type 2 MI should be designed to address specific phenotypes such as tachyarrhythmia, anemia, hypoxemia, or hypotension.

### study Limitations

There are several limitations which merit discussion. This is a small pilot phase randomized controlled trial which was not powered for statistical comparisons, and although we propose modification, no firm conclusions may be drawn as to the potential effectiveness of our intervention. While we screened consecutive patients with acute myocardial injury through electronic records to identify those with type 2 MI, this was an intensive task and delivery at scale would be challenging without sufficient resource. Although our trial was delivered across 3 sites, 96% of our patient population in Scotland identify as White Caucasian. Therefore, to ensure the results of any main phase trial are generalizable, this will require recruitment across more geographically and ethnically diverse populations. While we identified and approached similar numbers of eligible men and women to participate in the trial (31% vs 28%), fewer women agreed to be randomized (17% vs 12%, Supplemental Table 4), and most participants were male. Review of the screening logs demonstrated an excess of type 2 MI events in men identified and recruited during the last 2 months of the trial (Supplemental Figures 2 and 3). While this observation may reflect chance, women are consistently under-represented in cardiovascular trials, and ensuring balanced recruitment may require consideration at the trial design stage.^29^ We did not seek to recruit patients with type 2 MI due to coronary mechanisms as they typically present with ST-segment elevation and are managed as per conventional pathways for acute coronary syndrome. Finally, it is well described that there is significant heterogeneity in the investigation and treatment of patients with type 2 MI, and standard care observed within this pilot randomized controlled trial reflects local practice and may not be generalizable.

## Conclusions

TARGET-Type 2 has demonstrated it is feasible to prospectively identify, approach, recruit, and randomize patients with type 2 MI to a complex intervention of investigation and treatment for coronary heart disease and left ventricular impairment, but effective delivery will require adequate resources. This pilot phase trial has provided important insight into the potential effectiveness of our intervention which will inform the design of a main phase trial.

Perspectives **COMPETENCY IN PATIENT CARE 1:** Type 2 MI occurs due to myocardial oxygen supply or demand imbalance, without atherothrombosis, often during another illness. It is common and associated with poor clinical outcomes.

**COMPETENCY IN PATIENT CARE 2:** This pilot phase trial has demonstrated it is feasible to identify, recruit, and randomize patients with type 2 MI to a complex intervention targeting coronary and structural heart disease, with excellent adherence at 90 days.

**TRANSLATIONAL OUTLOOK:** A modified trial intervention mandating CT or invasive coronary angiography and echocardiography in all participants is likely to be more impactful and acceptable to clinicians and patients.

## Funding support and author disclosures

This work was supported by Chief Scientist Office (Scotland) TCS 22/31. Dr Taggart is supported by a Clinical Research Training Fellowship (FS/CRTF/21/2473) from the British Heart Foundation. Dr Boeddinghaus is supported by an Edinburgh Doctoral College Scholarship and research grants from the University of Basel, the University Hospital of Basel, the Division of Internal Medicine, the Swiss Academy of Medical Sciences, the Gottfried and Julia Bangerter-Rhyner Foundation, and the Swiss National Science Foundation. Drs Bularga and Wereski are supported by Clinical Research Training Fellowships (MR/V007254/1 and MR/V007017/1, respectively) from the Medical Research Council. Dr Williams is supported by the British Heart Foundation (FS/ICRF/20/26002). Dr Newby is supported by the British Heart Foundation (CH/09/002, RG/F/22/110093, and RE/18/5/34216) and was the recipient of a Wellcome Trust Senior Investigator Award (WT103782AIA). Dr Dweck is the recipient of the Sir Jules Thorn Award for Biomedical Science (15/JTA). Dr Mills is supported by a Chair Award (CH/F/21/90010), Programme Grant (RG/20/10/34966), and a Research Excellent Award (RE/24/130012) from the British Heart Foundation. All other authors have reported that they have no relationships relevant to the contents of this paper to disclose.
