# Supplementary material for: Targeting Investigation and Treatment in Type 2 Myocardial Infarction: A Pilot Randomized Controlled Trial
Source: JACC Adv. 2025 Apr 4;4(5):101738. doi: 10.1016/j.jacadv.2025.101738 (PMC12063109; doi:10.1016/j.jacadv.2025.101738)
Supplement: Supplementary data [file mmc1.docx]

**Supplemental Table 1:** Recruitment rate per site

|  | **Activation date** | **Total recruited (n=60)**  **N (%)** | **Total number of months in trial (N)** | **Monthly recruitment rate** |
| --- | --- | --- | --- | --- |
| **Royal Infirmary of Edinburgh** | 09/11/2022 | 49 (82) | 12.2 | 4 |
| **Western General Hospital, Edinburgh** | 14/12/2022 | 4 (6.7%) | 11 | 0.4 |
| **Victoria Hospital, Kirkcaldy** | 25/04/2023 | 7 (12) | 6.7 | 1.1 |

**Supplemental Table 2:** Physiological parameters

|  | **All patients (n=60)**  **N (%)** | **Intervention**  **(n=28)**  **N (%)** | **Standard care**  **(n=32)**  **N (%)** |
| --- | --- | --- | --- |
| **Physiological parameter** |  |  |  |
| *Heart rate (BPM)* | 135 (105-180) | 128 (98-150) | 140 (105-180) |
| *Systolic blood pressure (mmHg)* | 133 (92-170) | 129 (91-164) | 139 (94-201) |
| *Temperature (C)* | 36.7 (36.1-37.7) | 36.7 (36.2-37.3) | 36.5 (36.1-37.9) |
| *Respiratory rate (BPM)* | 20 (18-28) | 20 (18-36) | 20 (18-25) |
| *Oxygen saturation (%)* | 94 (88-98) | 93 (85-96) | 97 (93-99) |
| *On oxygen (Yes)* | 12 (20) | 6 (21) | 6 (19) |

**Supplemental Table 3:** Summary of qualitative interview findings

| **Theme** | **Subcategory** | **Evidence** |
| --- | --- | --- |
| Motivation for involvement | Altruism | *It was more altruistic than anything. Just being of assistance. If everyone said no you’d be up the spout* wouldn’t you? It was to be of use to somebody. If they could use my experience for the good of the trial that was fine.* (Interview Participant 15)  **’Up the spout’* (informal British) – wasted, spoiled or not working correctly |
|  | Additional care | *It means you’ll be monitored, which is a good thing, but it also means that you’ll be helping clinicians get to, to come to new solutions to a very common problem. The monitoring, I think was the clincher, it was the thing that made me really feel, well, yeah, this is great thing to do and it’s going to be good for me as well as for the research team.* (Interview Participant 5) |
|  | Others promoting involvement | *I had a conversation with a good friend who’s a GP that I talked with. But, I mean, I’d already made the decision to take part and that just confirmed that this was a good thing to do.* (Interview Participant 5) |
| Process of enrolment and randomisation | Method of approach | *I was actually quite excited [to get the phone call], I mean, this was a strange thing to happen and so, yes, I thought, how good, the health service are doing something about it. So, yes, I was very pleased.* (Interview Participant 1) |
|  |  | *I came off the phone and said to my husband, ‘they said I got a heart attack!’ and I burst into tears. I would have felt safer in a hospital setting where I could ask a nurse or the research team. I think the sheet [patient information sheet] does say you’ve not had a heart attack but by that time the alarm bells are off.* (Focus Group Participant 2) |
|  | Equipoise | *I was happy with the care I received. I didn’t feel the need for anything extra. No disappointment at all. It was a good outcome.* (Interview Participant 15 randomised to standard care) |
|  |  | *I suppose, in one respect, I felt an element of relief because I thought, right, okay, I’ve done my duty, I’ve decided to turn up for this trial, and it hasn’t really cost me anything because I would have been going this way anyway. You know, so there was an element of relief with that. I suppose when I was talking to you earlier on about the more selfish aspect of it, I thought, oh, well, nobody’s going to take a particular interest in me, here. I’m just going to be rolling along with everybody else. So probably both feelings, to be honest, one of relief and one of, sort of, slight disappointment.*(Interview Participant 9 randomised to standard care) |
|  |  | *When you hear some of them going on trials and it is giving them a bit longer because of the drugs they are taking, that is how I immediately saw that one, I thought, oh, I am going to miss out on something.* (Interview Participant 8 randomised to standard care) |
|  | Understanding the intervention | *Initially, when I had a look at it, I thought the trial was going to involve, sort of, angiograms and different types of processes which I just felt uncertain about just exactly what would be put into my blood stream and I wasn’t too comfortable about that. Therefore, I decided, no, the best way to do this is just to decide that I wasn’t going to take part in it.* (Interview Participant 2) |
| Understanding diagnosis | Clinical use of terminology | *But when I was in hospital, even when I was speaking to the cardiologist nobody mentioned a type 2 MI or anything that made you think it could have an ongoing effect in my heart. It [discharge letter] said AF with associated troponin rise.* (Focus Group Participant 2) |
|  | Patient processing of information | *I first went to hospital A&E with atrial fibrillation. And, so they said there was some enlargening (sic) of one of the chambers, the ventricles, maybe, but they said that was normal of atrial fibrillation. I’m not sure if that is what myocardial infarction is. But, umm, I have done some research, having heard the term but I am still, am not really any of the wiser in terms of how it specifically relates to my problems.* (Focus Group Participant 1) |
|  | Emotional impact of unexpected diagnosis | *When they told me I had a problem with my heart that frightened me.* (Focus Group Participant 4) |
|  | Practical impact of diagnosis | *Can I ask what terms do insurance companies recognise? That was one of the reasons I initially didn’t want to be in the trial because I thought I’m going to have to declare that. So that’s good to know because now I’ll have to do that.* *I’ve probably had that before because I’ve had the heart thing and not gone to hospital. It’s just because I was there and someone measured the troponin. If having that on your record impacts your holiday insurance but doesn’t give you any extra care, or information, or access to help, what’s the point? Now it’s there it’s there.* (Focus Group Participant 2) |
|  | Access to information regarding diagnosis and prognosis | *As long as you stay away from Google cos it tells you that – I’m not even going to say the word. You’ve all read it. I don’t want my children reading it. That worries me more, what they think and what they are worried about more than anything I’m going through. It’s worst-case scenarios isn’t it. You’ve got to stay away from it.* (Focus Group Participant 5) |
|  |  | *We were not told what to expect, what will happen next. I’m older than most of you- nobody has said, you know, stop doing.., you can expect to be ok for the next 5 years, and maybe you can go downhill, and it’s important for plans, grandchildren and all the rest of it. (*Focus Group *Participant 3)* |
| Reasons for non-participation | Lack of understanding | *Because my granddaughter says, what’s the difference between AF and then going into this thing [type 2 myocardial infarction]. And she couldn’t see the connection. (Interview Participant 3)* |
|  | Co-morbidity burden | *I’d got several things wrong with me, at the time. I mean, I’ve got colitis, I’ve got COPD. I mean, well, I think they were examining me for long COVID. I had a big operation … and that’s been giving me bother recently. So, I went for a scan for that, a few weeks ago. So, I’m just waiting and to see what’s going to happen with that. I don’t really want to think about anything else.* (Interview Participant 7) |
|  | Additional research activity | *I mean when I was approached about it, I mean I think I’d already been approached by two different research groups. I don’t know if you’re aware of that.* (Interview Participant 2) |
|  | Feeling dismissed by clinical team | *They told me there’s nothing wrong with me. There’s no need if there’s nothing wrong.* (Interview Participant 9) |

**Supplemental Table 4:** Proportion of men and women screened, eligible, approached, consented and randomised to the complex intervention or standard care

|  | **Screened** | **Eligible**  **N (%)** | **Approached**  **N (%)** | **Consented**  **N (%)** | **Randomised**  **N (%)** |
| --- | --- | --- | --- | --- | --- |
| **Female** | 185 | 61 (33) | 52 (28) | 23 (12) | 23 (12) |
| **Male** | 218 | 82 (38) | 67 (31) | 37 (17) | 37 (17) |


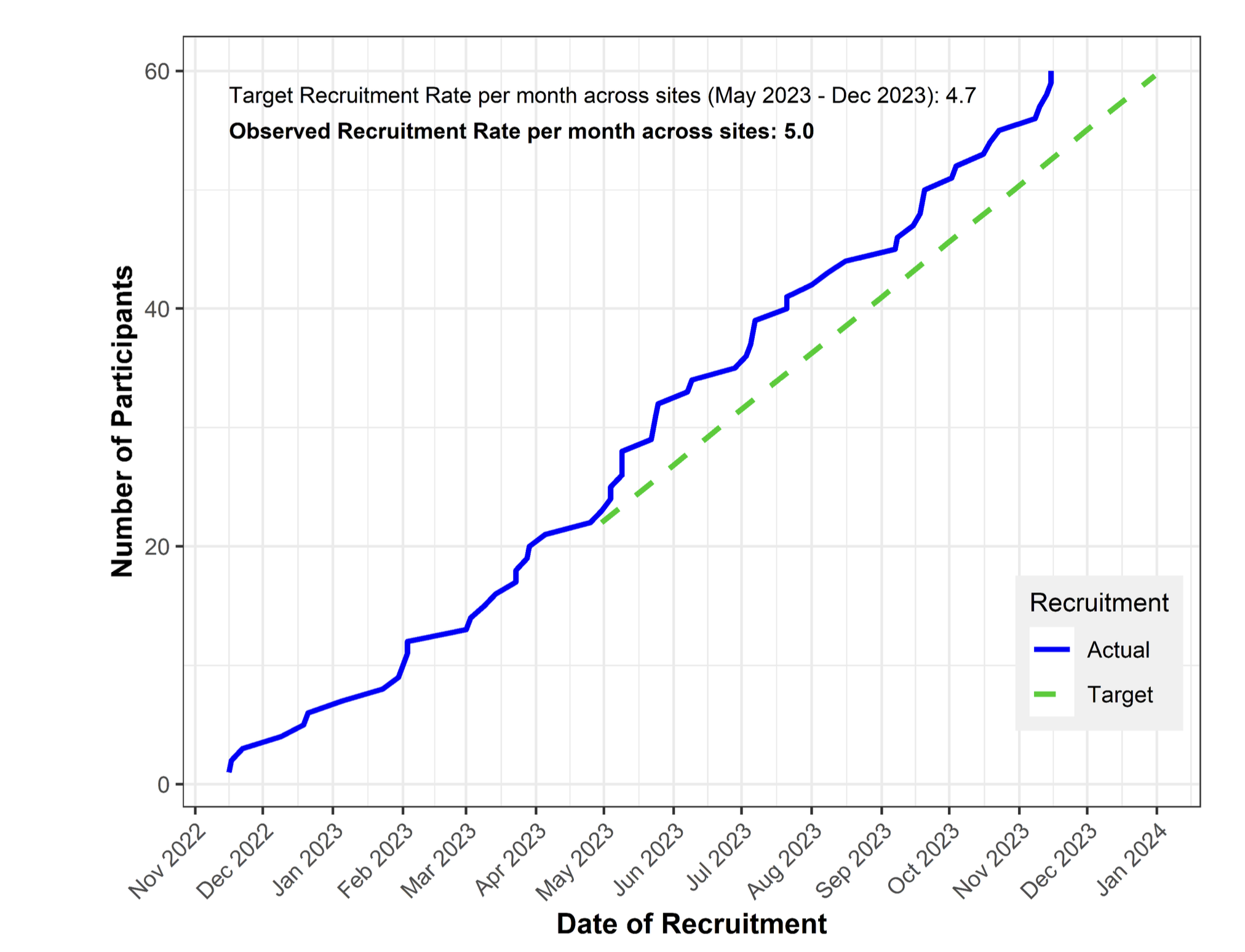


**Supplemental Figure 1:** Observed versus planned recruitment rate.

**
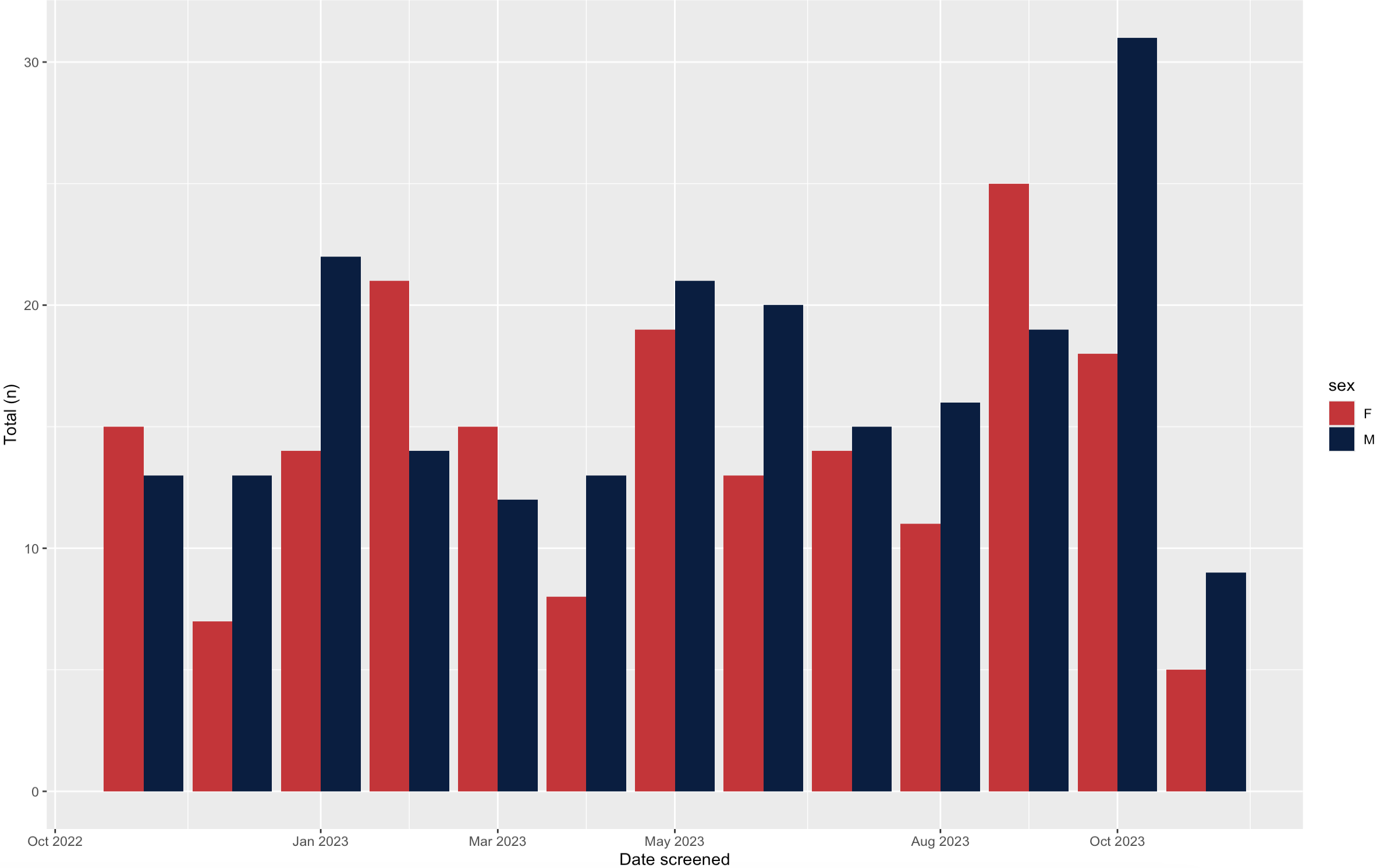
**

**Supplemental Figure 2:** Number of men and women screened per month of trial

**
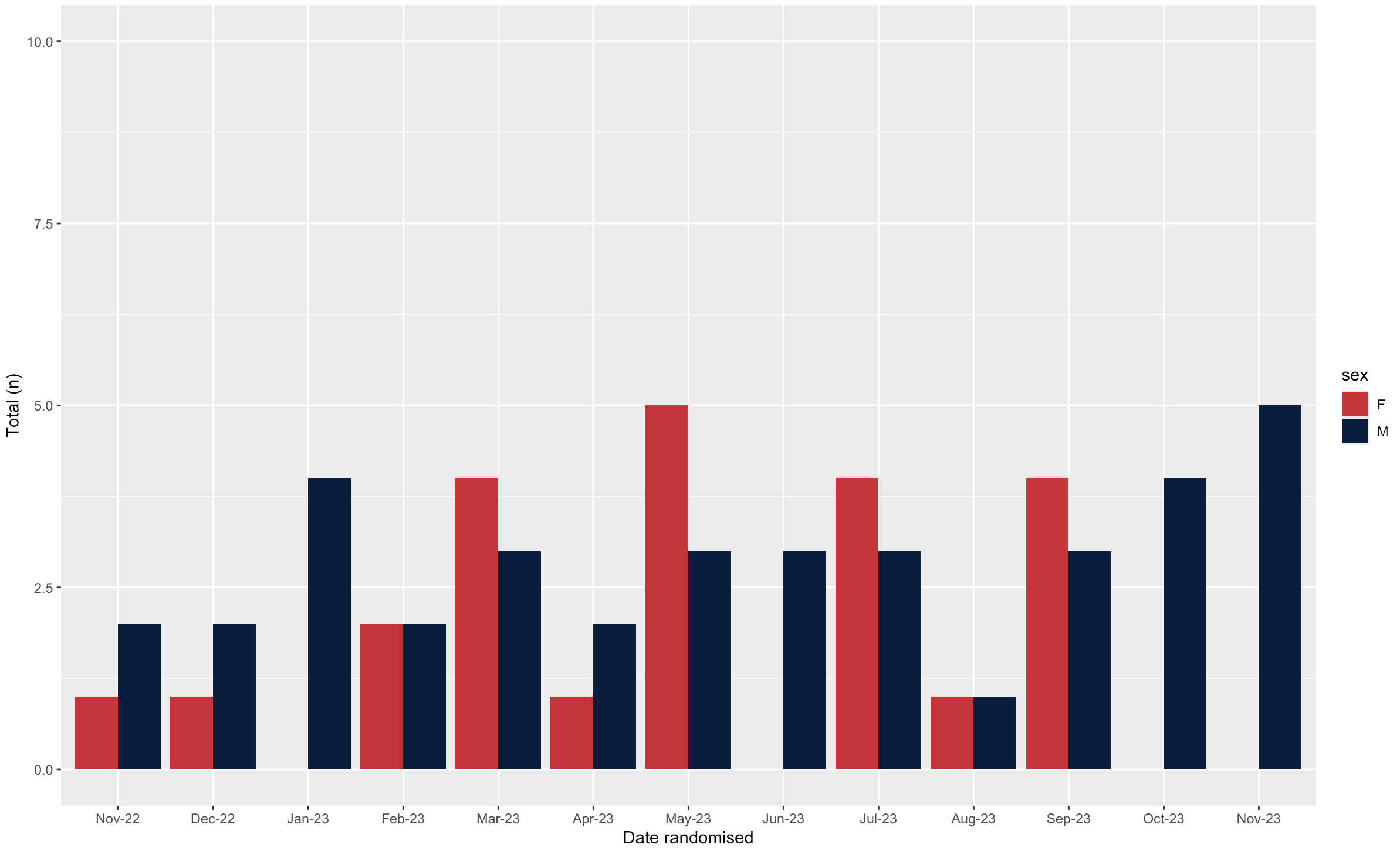
**

**Supplemental Figure 3:** Number of men and women randomised per month of trial

**Supplemental Appendix**

Flow diagram demonstrating recommended investigational strategy and communication of treatment recommendations to the usual clinical team.


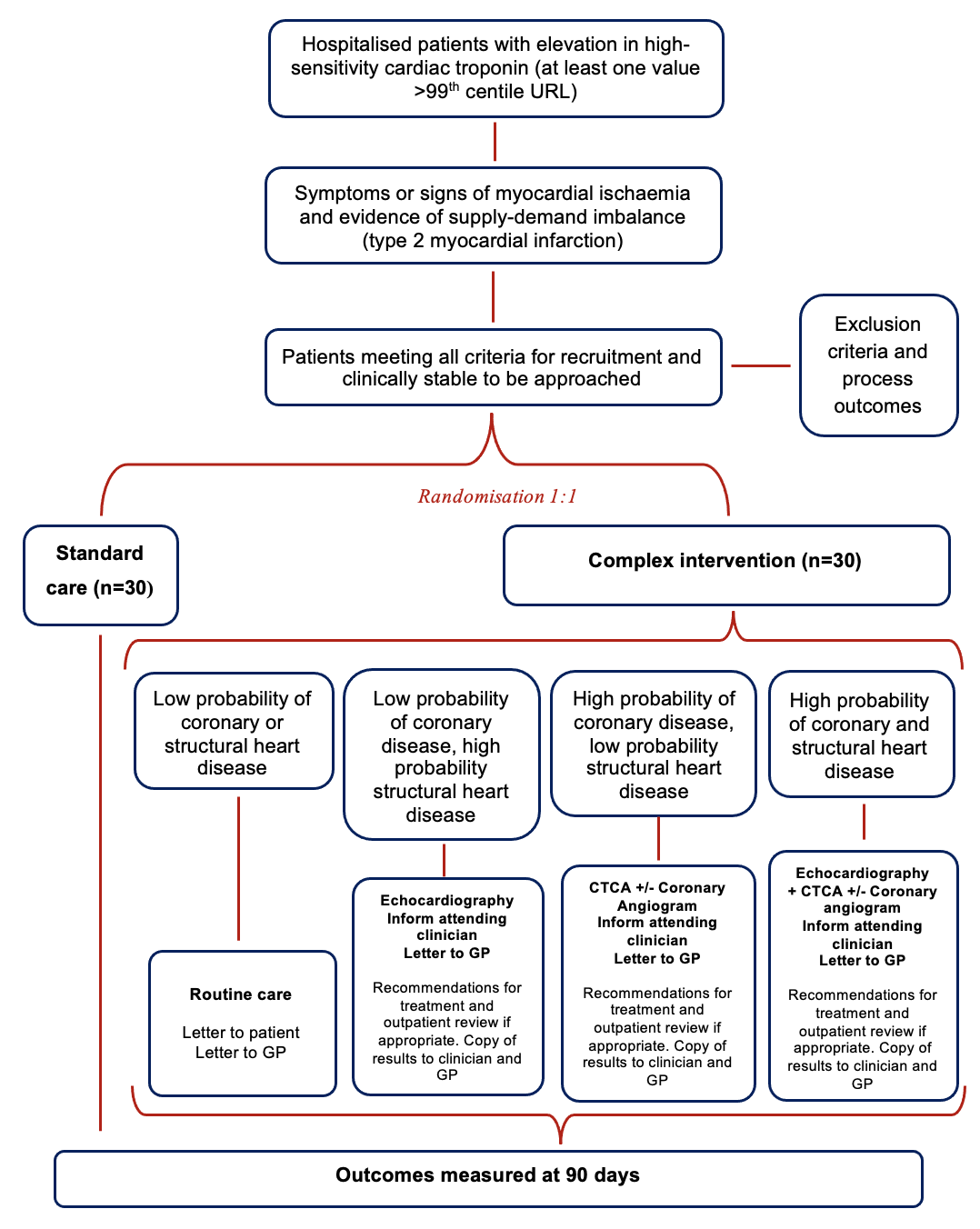


**Supplemental Appendix**

**TARGET Type 2 Trial Steering Committee Members**

**Independent Chairperson:** Professor Paul Collinson

**Independent members**: Professor Stephen Leslie, Professor Peter Craig

**Statistician**: Professor Steff Lewis, Dr Anny Briola

**Co-applicants**: Dr Amy Ferry, Professor Rustam Al-Shahi Salman, Professor Nicholas Mills

**Independent lay person**: Redacted

**Trial Program Manager:** Mr Chris Tuck
**Principal Investigator (Lothian) and Facilitator:** Dr Caelan Taggart

**Chief investigator:** Dr Andrew Chapman

**Detailed description of diagnostic adjudication.**

All patients with hs-cTnI concentrations above the sex-specific 99th centile were classified according to the Fourth Universal Definition of Myocardial Infarction. Two physicians independently reviewed all clinical information with discordant diagnoses resolved by a third reviewer. Clinical information included the dates and times of presentation and final discharge, the initial emergency department assessment and final discharge letter as documented in the electronic care record, with summaries of all investigations undertaken during the index presentation including the electrocardiogram. The adjudication panel had access to raw clinical information including haemoglobin, creatinine and high-sensitivity cardiac troponin I concentrations, and the reports from invasive coronary angiography. Type 1 myocardial infarction was defined as myocardial necrosis (any hs-cTnI concentration above the 99th centile with a rise and/or fall in hs-cTnI concentration where serial testing was performed) in the context of a presentation with suspected acute coronary syndrome with symptoms or signs of myocardial ischemia on the electrocardiogram. Patients with symptoms or signs of myocardial ischemia and evidence of increased oxygen demand or decreased supply (for example, tachyarrhythmia, hypotension, or anaemia) secondary to an alternative pathology and myocardial necrosis were defined as type 2 myocardial infarction. The classification of type 2 myocardial infarction also includes patients with coronary vasospasm, embolism or spontaneous dissection without evidence of atherothrombosis related to coronary artery disease. Type 4a myocardial infarction was defined in patients with symptoms or signs of myocardial ischemia following percutaneous coronary intervention where hs-cTnI concentrations were 5-fold greater than the 99th centile, or increased further if elevated prior to the procedure. Type 4b myocardial infarction was defined where myocardial ischemia and myocardial necrosis were associated with stent thrombosis documented at angiography. Myocardial injury was defined if hs-cTnI concentrations were above the 99th centile in the absence of any clinical features of myocardial ischemia. All non-ischemic myocardial injury was classified as acute, unless a change of <20% was observed on serial testing or the final adjudicated diagnosis was chronic heart failure or chronic renal failure, where the classification was chronic myocardial injury.

**TARGET-Type 2 Interview topic guide – patient**

As part of this trial, we would like to understand more about the reasons patients agree or decline to participate in the trial.

Thank participant for agreeing to take part in an interview.

Interviews will be led by topics important to the patient, but potential topics include:

**Topic 1- Presentation of trial information**

Could you talk about how the trial information was presented to you?
Was the timing appropriate?
Did you consent to the trial in hospital or over the phone after discharge?

Did the information sheet explain what the trial is aiming to achieve adequately?

Did anything need additional clarification from a researcher or member of your clinical team?

**Topic 2 - Understanding of study processes**

Do you have any comment on the randomisation process (allocation to study group)?

If consented - How did you feel when you were allocated to standard care/ the intervention arm?

For those in intervention arm – can you tell me about the additional tests you had done as part of the study and how you felt about that?

**Topic 3 - Decision to consent or decline**
Could you talk a bit more about how and why you made the decision to consent/decline the trial?

How easy did you find the decision of whether to take part or not?
